# Incidence of coronary heart disease among remote workers: a nationwide web-based cohort study

**DOI:** 10.1038/s41598-024-59000-y

**Published:** 2024-04-10

**Authors:** Masayoshi Zaitsu, Tomohiro Ishimaru, Saki Tsushima, Keiji Muramatsu, Hajime Ando, Tomohisa Nagata, Hisashi Eguchi, Seiichiro Tateishi, Mayumi Tsuji, Yoshihisa Fujino

**Affiliations:** 1 Center for Research of the Aging Workforce, University of Occupational and Environmental Health, Japan, Kitakyushu, Japan; 2 Department of Medical Humanities, School of Medicine, University of Occupational and Environmental Health, Japan, Kitakyushu, Japan; 3 Department of Preventive Medicine and Community Health, School of Medicine, University of Occupational and Environmental Health, Japan, Kitakyushu, Japan; 4 Department of Work Systems and Health, Institute of Industrial Ecological Sciences, University of Occupational and Environmental Health, Japan, Kitakyushu, Japan; 5 Department of Occupational Health Practice and Management, Institute of Industrial Ecological Sciences, University of Occupational and Environmental Health, Japan, Kitakyushu, Japan; 6 Department of Mental Health, Institute of Industrial Ecological Sciences, University of Occupational and Environmental Health, Japan, Kitakyushu, Japan; 7 Disaster Occupational Health Center, Institute of Industrial Ecological Sciences, University of Occupational and Environmental Health, Japan, Kitakyushu, Japan; 8 Department of Environmental Health, School of Medicine, University of Occupational and Environmental Health, Japan, Kitakyushu, Japan; 9 Department of Environmental Epidemiology, Institute of Industrial Ecological Sciences, University of Occupational and Environmental Health, Japan, 1-1 Iseigaoka, Yahata-Nishi-Ku, Kitakyushu, Fukuoka 807-8555 Japan

**Keywords:** Socioeconomic scenarios, Risk factors, Environmental social sciences, Acute coronary syndromes, Epidemiology

## Abstract

Coronary heart disease (CHD) risk is influenced by socioeconomic status-related parameters, particularly occupation. We investigated occupational gaps in CHD risk and how the introduction of remote work moderated the observed occupational differences in CHD risk during the coronavirus disease 2019 pandemic in Japan. Data from a web-based, nationwide cohort study, comprising 17,640 workers (aged 20–65 years) with baseline data from December 2020, were analyzed. Participants were grouped by occupation as upper-level nonmanual workers (managers/professionals) and others (reference group). The primary outcome was CHD (angina pectoris/myocardial infarction) onset retrospectively confirmed at the 1-year follow-up survey. Upper-level nonmanual workers exhibited a higher CHD incidence than others (2.3% vs. 1.7%). This association was pronounced in the younger (20–49 years) population, with a significant CHD risk (adjusted risk ratio = 1.88). Upper-level nonmanual workers exhibited nearly 15% higher remote work prevalence, with a significant remote work-related CHD risk (adjusted risk ratio = 1.92). The mediating effects of remote work explained an overall disparity of 32% among the younger population. Occupational gaps in CHD incidence in Japan differ from those in Western countries, where upper-level nonmanual workers have lower cardiovascular risk. In Japan, remote work can mediate CHD risk in the younger population of upper-level nonmanual workers.

## Introduction

Coronary heart disease (CHD) is a leading cause of morbidity and mortality worldwide, with significant emphasis on the fundamental influence of socioeconomic status (SES)-related parameters, particularly occupation, on CHD risk^1,2^. Numerous studies have extensively documented contemporary, universal occupational gaps in CHD risk^3,4^, highlighting an “inverse” pattern: individuals in higher occupational classes, such as those holding upper-level nonmanual jobs, including managerial and professional positions, tend to experience lower cardiovascular risk than do their counterparts in lower occupational strata^5,6^. Occupational factors, such as job-related stress and sedentary work, and established behavioral risks, such as smoking and elevated serum cholesterol levels, have been identified as mediators of occupational CHD risk^4,7,8^.

However, occupational disparities in CHD risk have remained understudied in Japan, and some previous studies have reported associations deviating from those observed in Western countries^6,9–11^. Prior nationwide studies in Japan have shown a “positive” pattern, where individuals in higher occupational classes exhibited an increased CHD risk^11^. These findings challenge conventional knowledge, highlighting that occupational gaps in CHD risk may be diverse even in developed countries with high-quality medical care accessible to all through universal health coverage.

Furthermore, the prominence of remote work, particularly during the coronavirus disease 2019 (COVID-19) pandemic, has been a focus area in terms of occupational health^12^. Recent studies have revealed the psychological distress associated with remote work^13–15^. However, the impact of this trend on occupational CHD disparities remains unexplored. As Japan confronted the challenges of the pandemic, remote work became a pivotal aspect of employment practices (> 60% of companies adopted remote work)^13^. Nonetheless, the extent to which remote work, a hallmark of the COVID-19 era, may influence these risks remains uninvestigated.

Therefore, this study aimed to examine occupational gaps in CHD risk in Japan to elucidate how the emergence of remote work, a novel working style in Japan, mediated the observed occupational disparities in CHD risk during the COVID-19 pandemic. Here, we show for the first time that the benefits to cardiovascular health experienced by upper-level nonmanual workers may not always be universally applicable in remote work.

## Results

Table 1 displays the baseline characteristics and outcomes of the study cohort. The incidence of CHD was higher among CHD among upper-level nonmanual workers than among their counterparts in other-occupation categories (2.3% vs. 1.7%). This disparity in CHD incidence was particularly pronounced in the younger population (3.1% vs. 1.3%), whereas no significant difference was observed in the older population. Upper-level nonmanual workers exhibited nearly 15% and 10% higher prevalence for remote work and smoking, respectively, than did the other-occupation groups. Additionally, among 4536 current smokers, the proportion of those who had increased their smoking habits at baseline was higher among remote workers than among non-remote workers (15.2% vs. 9.7%; Table 2). Meanwhile, the prevalence of long working hours did not differ between non-remote and remote workers: 1142/13728 (8.3%) versus 339/3912 (8.7%, *P* = 0.49), respectively.

**Table 1:** Overall and age-stratified baseline characteristics and outcomes.

Characteristics	N (%) or mean (SD)		*P*-value^a^
	Others	Upper-level nonmanual	
Entire population (age 20–65 years)			
N	15,754	1886	
Incident CHD	265 (1.7%)	43 (2.3%)	0.06
Remote work at baseline	3249 (20.6%)	663 (35.2%)	< 0.001
Female	7321 (46.5%)	260 (13.8%)	< 0.001
Age, mean (SD)	47.9 (10.0)	53.3 (7.2)	< 0.001
High school or less	4495 (28.5%)	284 (15.1%)	< 0.001
Low household income < 2 million JPY	1041 (6.6%)	31 (1.6%)	< 0.001
Ever smoker	7350 (46.7%)	1190 (63.1%)	< 0.001
Former smoker	3166 (20.1%)	518 (27.5%)	< 0.001
Current smoker	4184 (26.6%)	672 (35.6%)	
Habitual drinker > 1 day/week	6523 (41.4%)	1100 (58.3%)	< 0.001
Physically active	6293 (39.9%)	833 (44.2%)	< 0.001
Body mass index, mean (SD)	22.4 (3.7)	23.3 (3.4)	< 0.001
Hypertension	1511 (9.6%)	325 (17.2%)	< 0.001
Diabetes	561 (3.6%)	108 (5.7%)	< 0.001
Kessler 6 scores > 10	2746 (17.4%)	196 (10.4%)	< 0.001
Weekly working hours > 55 h	1280 (8.1%)	201 (10.7%)	< 0.001
Younger population (20–49 years)			
N	8185	514	
Incident CHD	108 (1.3%)	16 (3.1%)	< 0.001
Remote work at baseline	1460 (17.8%)	172 (33.5%)	< 0.001
Female	5036 (61.5%)	159 (30.9%)	< 0.001
Age, mean (SD)	40.1 (7.0)	44.0 (4.9)	< 0.001
High school or less	2042 (24.9%)	64 (12.5%)	< 0.001
Low household income < 2 million JPY	517 (6.3%)	5 (1.0%)	< 0.001
Ever smoker	2980 (36.4%)	246 (47.9%)	< 0.001
Former smoker	1197 (14.6%)	94 (18.3%)	< 0.001
Current smoker	1783 (21.8%)	152 (29.6%)	
Habitual drinker > 1 day/week	2737 (33.4%)	257 (50.0%)	< 0.001
Physically active	3140 (38.4%)	231 (44.9%)	0.003
Body mass index, mean (SD)	21.8 (3.6)	22.8 (3.8)	< 0.001
Hypertension	274 (3.3%)	31 (6.0%)	0.001
Diabetes	111 (1.4%)	10 (1.9%)	0.27
Kessler 6 scores > 10	1797 (22.0%)	77 (15.0%)	< 0.001
Weekly working hours > 55 h	597 (7.3%)	69 (13.4%)	< 0.001
Older population (50–65 years)			
N	7569	1372	
Incident CHD	157 (2.1%)	27 (2.0%)	0.80
Remote work at baseline	1789 (23.6%)	491 (35.8%)	< 0.001
Female	2285 (30.2%)	101 (7.4%)	< 0.001
Age, mean (SD)	56.4 (4.3)	56.7 (4.2)	0.004
High school or less	2453 (32.4%)	220 (16.0%)	< 0.001
Household income < 2 million JPY	524 (6.9%)	26 (1.9%)	< 0.001
Ever smoker	4370 (57.7%)	944 (68.8%)	< 0.001
Former smoker	1969 (26.0%)	424 (30.9%)	< 0.001
Current smoker	2401 (31.7%)	520 (37.9%)	
Habitual drinker > 1 day/week	3786 (50.0%)	843 (61.4%)	< 0.001
Physically active	3153 (41.7%)	602 (43.9%)	0.13
Body mass index, mean (SD)	23.0 (3.6)	23.5 (3.3)	< 0.001
Hypertension	1237 (16.3%)	294 (21.4%)	< 0.001
Diabetes	450 (5.9%)	98 (7.1%)	0.09
Kessler 6 scores > 10	949 (12.5%)	119 (8.7%)	< 0.001
Weekly working hours > 55 h	683 (9.0%)	132 (9.6%)	0.48

^a^*P*-values for t-test or Chi-square test.

**Table 2:** Prevalence of current smokers reporting increased smoking quantity at baseline compared to that before the COVID-19 pandemic.

Characteristics	N (%)		*P*-value^a^
	Non-remote worker	Remote worker	
Entire population			
N	3758	1098	
Increased smoking quantity	341 (9.7%)	154 (15.2%)	< 0.001
Younger population			
N	1566	369	
Increased smoking quantity	199 (13.4%)	68 (19.7%)	0.003
Older population			
N	2192	729	
Increased smoking quantity	142 (6.9%)	86 (13.0%)	< 0.001

^a^*P*-values for Chi-square test.

**Figure 1:**
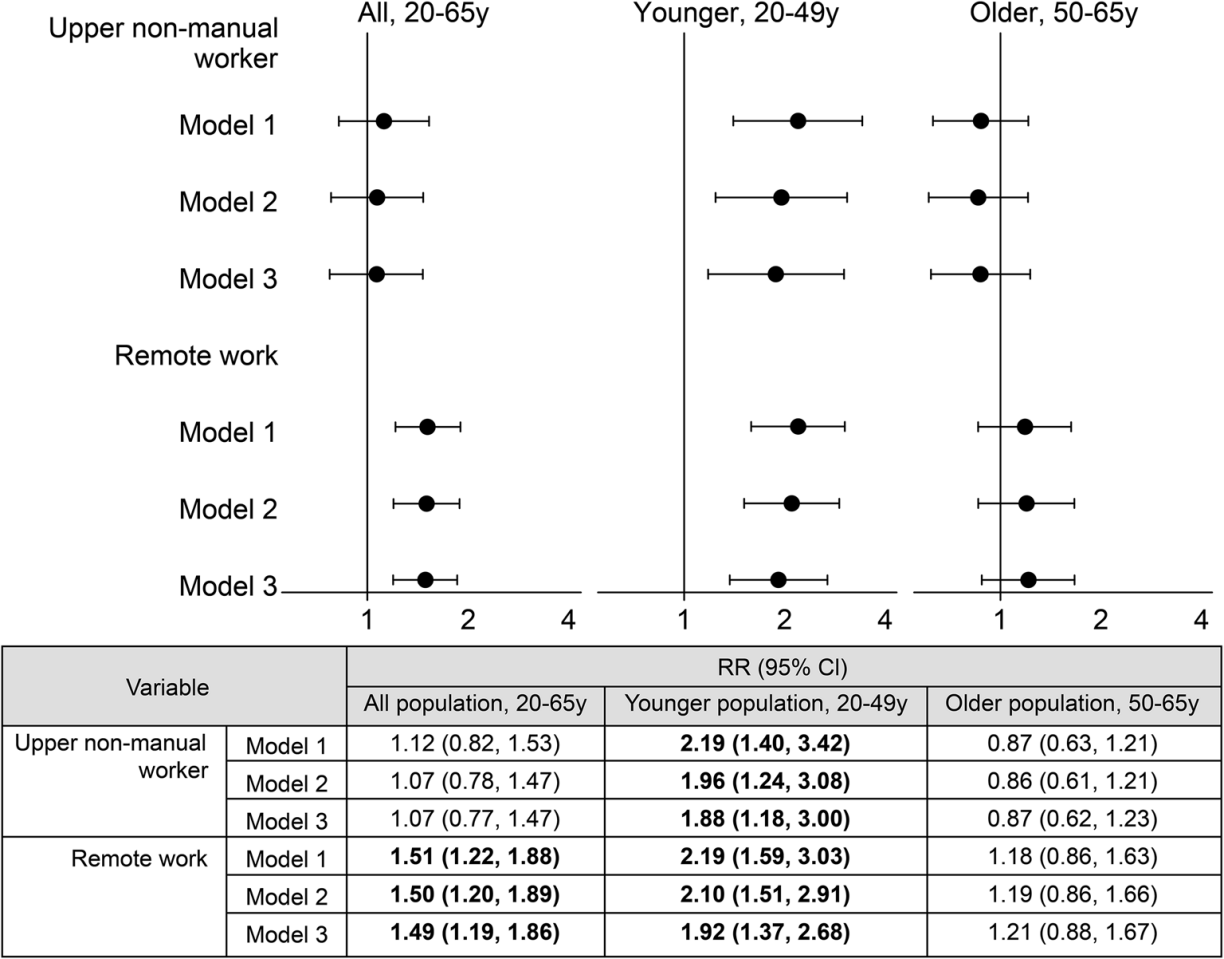
Relative risks and 95% confidence intervals for coronary heart disease incidence Relative risks were estimated using two-level multilevel Poisson regression with robust variance. Participants (Level 1) were nested within 47 prefectures (Level 2), and a random intercept was employed for prefectures. Adjustments were made for basic confounding variables of sex, age, educational attainment, and household income in Model 1. In Model 2, covariates simultaneously included upper-level nonmanual workers and remote workers. In Model 3, further adjustments were made for the following potential mediating variables: behavioral factors (smoking, heavy drinking, and physical activity), clinical factors (body mass index, hypertension, and diabetes), psychological distress, and long working hours. Abbreviations: RR, Relative risk; CI, confidence interval.

The relative risks (RRs) for CHD incidence associated with occupation are presented in Fig. 1 (see full results for other variables in Supplemental Table S1). While the RR of upper-level nonmanual workers did not reach statistical significance in the overall and older populations, it demonstrated a significant increase in the younger population (Model 1: RR = 2.19, 95% CI 1.40–3.42). After adjusting for remote work in Model 2, the RR was attenuated but remained significant (RR = 1.96, 95% CI 1.24–3.08). Finally, in Model 3, with full adjustments for behavioral, clinical, mental, and occupational risks, the RR was significant (RR = 1.88, 95% CI 1.18–3.00). The remote work RR for CHD incidence was not elevated in the older population; however, it was significantly elevated in the overall and younger populations (RR = 1.49, 95% CI 1.19–1.86 and RR = 1.92, 95% CI 1.37–2.68, respectively; Fig. 1). The RR of hypertension was elevated while that of long working hours was not significant (Supplemental Table S1).

Lastly, in the mediation analysis of the association between occupation and CHD incidence among the younger population (for all and older populations, see Supplemental Table S2), the mediating effects of remote work explained an overall proportion of 32% (Table 3). Sex-specific analyses showed similar patterns, while the impact was most pronounced in younger men (Table 4).

**Table 3:** Results of causal mediation analysis and four-way decomposition among younger workers aged 20–49 years.

Four-way decomposition	Coefficient (95% CI)
Total excess odds ratio^a^	1.20 (− 0.03–2.43)
Excess odds ratio due to CDE	0.42 (− 0.66–1.50)
Excess odds ratio due to INTref	0.39 (− 0.15–0.94)
Excess odds ratio due to INTmed^b^	0.28 (− 0.12–0.69)
Excess odds ratio due to PIE^b^	0.11 (0.02–0.19)
Total effect	2.20 (0.97–3.43)
Proportion CDE	0.35 (− 0.36–1.06)
Proportion INTref	0.33 (− 0.08–0.73)
Proportion INTmed	0.24 (− 0.06–0.53)
Proportion PIE	0.09 (− 0.02–0.20)
Overall proportion mediated	0.32 (0.00–0.65)
Overall proportion attributable to interaction	0.56 (− 0.13–1.25)
Overall proportion eliminated	0.65 (− 0.06–1.36)

CDE, controlled direct effect; INTref, reference interaction; INTmed, mediated interaction; PIE, pure indirect effect; CI, confidence interval.

^a^Total excess odds ratio for CHD = Excess odds ratio due to CDE + Excess odds ratio due to INTref + Excess odds ratio due to INTmed + Excess odds ratio due to PIE.

^b^Total indirect effect = PIE + INTmed.

**Table 4:** Sex-specific relative risks of coronary heart disease incidence in upper-level non-manual workers.

Variable	Relative risk (95% confidence interval)^a^		
	Entire, 20–65 y	Younger, 20–49 y^b^	Older, 50–65 y
Men			
Upper-level nonmanual	1.04 (0.77, 1.39)	1.84 (1.12, 3.04)	0.89 (0.64, 1.23)
Remote work	1.40 (1.06, 1.85)	1.63 (1.17, 2.28)	1.27 (0.87, 1.84)
Women			
Upper-level nonmanual	1.42 (0.45, 4.45)	1.98 (0.61, 6.42)	0.86 (0.18, 4.01)
Remote work	1.73 (1.00, 3.00)	2.21 (1.30, 3.75)	0.86 (0.30, 2.44)

^a^Sex-specific relative risks were estimated using two-level multilevel Poisson regression with robust variance. Adjustments were made for basic confounding variables, remote workers, behavioral factors, clinical factors, psychological distress, and long working hours.

^b^Overall proportion mediated for the younger population was 0.32 (95% CI − 0.06 to 0.69) in male workers and 0.38 (95% CI − 0.34 to 1.10) in female workers.

## Discussion

In this study, we examined occupational gaps in CHD risk in Japan to elucidate how the emergence of remote work mediated the observed occupational disparities in CHD risk during the COVID-19 pandemic. Contrary to the SES gap in CHD observed in many developed countries, where higher occupational classes typically exhibit lower CHD risks, the present study confirmed that individuals in higher occupational classes in Japan tended to face higher CHD risks during the COVID-19 pandemic. Individuals in higher occupational groups faced nearly double the risk of CHD incidence among younger men. This aligns with the results of previous studies conducted in this country^6,9–11^. Interestingly and unexpectedly, this study also made a novel observation that remote work appears to play a role in “increasing” cardiovascular risk. Thus, it emerges as a significant mediator for occupation-related CHD incidence in Japan, accounting for approximately one-third of the mediating effects.

These deviations from the contemporary SES pattern may partly be attributed to behavioral risk factors, notably smoking, associated with remote-site workplaces, including the home, which is physically outside of the original workplace. In Japan, considerable efforts have been invested in promoting smoking cessation as part of occupational health practices. However, this study revealed that the prevalence of current smoking is possibly higher in upper-level nonmanual workers than in other-occupation groups. When these upper-level nonmanual workers engage in remote work, often from their homes, the smoking regulations and policies of their original workplace no longer apply. Consequently, quitting smoking becomes more challenging^16^, and it is conceivable that the quantity of smoking may even increase under these circumstances, as observed among current smokers in the present study.

In some instances, remote work has been reported to increase workers’ productivity and be associated with better mental health^17^. However, current occupational health research has focused on the fact that social support from coworkers tends to decrease among remote workers, resulting in occupational distress^13–15,18^. During the present study period, when remote work had rapidly spread in its early stage in Japan, younger managers and professionals may have faced increased workloads without adequate social support, given that remote work was a new working style that was not experienced previously. Therefore, the subsequent job stress may have contributed to an increased CHD risk among this specific population. In addition, a higher prevalence of longtime working among upper-level nonmanual workers may have influenced CHD in relation to job stress in this study.

This study has some limitations. First, despite being conducted in a cohort design, CHD onset was retrospectively self-reported at the 1-year follow-up point, which was not enough to sufficiently observe CHD onset. Consequently, detailed clinical diagnoses and information were unavailable for analysis. Selection bias was inevitably introduced in this Internet cohort study, as respondents self-selected for participation; thus, the generalizability was partly limited. However, the follow-up rate of almost 70% was fair. Second, the regression analyses comprehensively considered numerous multi-dimensional variables, including behavioral, clinical, mental, and occupational factors. However, the intensity of smoking and the impact of heated tobacco products, which are new tobacco products that have gained popularity during the study period^19^ could not be assessed owing to a lack of information. Unadjusted confounding variables may have also been present. Nevertheless, the study’s strength lies in its findings that shed light on remote work in combination with occupational class, which has become a “new normal” among younger generations but presents disadvantages in terms of cardiovascular health. These novel findings may help elucidate the underlying mechanisms of occupational disparities in CHD related to remote work in future studies.

In conclusion, this study reveals that the current occupational disparities in CHD incidence in Japan follow a pattern different from that in Western countries. Among the younger population, remote work appears to mediate coronary risk for upper-level nonmanual workers. These findings highlight the complex interplay of occupational factors in CHD risk and call for further investigation into the impact of remote work on cardiovascular health within different demographics.

## Methods

### Study design and setting

This nationwide, web-based cohort study followed 17,640 participants over the course of 1 year. Data were extracted from the Collaborative Online Research on the Novel-coronavirus and Work (CORoNaWork) Project, an extensive online cohort survey of Japanese workers enrolled in December 2020 during the country’s “third wave” of COVID-19 infections^20^. Details of the baseline survey and follow-up protocol have been described^20–22^. In brief, the study cohort encompassed individuals who were workers aged 20–65 years at baseline. They were selected via a systematic sampling strategy that considered factors such as sex, occupation, and regional residence within Japan. This study was performed in accordance with the Declaration of Helsinki and was approved by the Ethics Committee of the University of Occupational and Environmental Health, Japan (no. R2-079). Informed consent was obtained.

From an initial recruitment pool of 33,087 participants, 6051 participants who provided invalid responses were excluded, retaining 27,036 eligible participants for the baseline analysis of the CORoNaWork Project (Wave 1). Subsequently, in this cohort study, 18,560 participants were respondents to a 1-year follow-up survey in December 2021 (response rate, 68.6%; Wave 2). Among these, 920 participants with a medical history of CHD at baseline were excluded (see profiles in Supplemental Table S3), yielding the analytical cohort of 17,640 working individuals aged 20–65 years who had never experienced CHD at the baseline.

### Primary outcome: CHD onset

The primary outcome was CHD onset, which was retrospectively confirmed at Wave 2. The question about CHD incidence in the Wave 2 survey was phrased as follows: “Are you presently undergoing outpatient care or treatment for angina pectoris or myocardial infarction?” Participants responded with one of the following options: “I have never had this illness,” “I am currently undergoing outpatient care or treatment,” “I was previously receiving outpatient care or treatment, but I am currently on self-interruption,” “I was advised to undergo tests or treatment, but I have not visited a medical institution,” or “I am currently cured or in remission (no longer requiring treatment).”

The absence of CHD during the follow-up period was defined as a response of “I have never had this illness.” Meanwhile, the other four responses indicated incident CHD during the study period. The exact date of CHD onset was not available in this study.

### Occupational class

For occupational class, participants were grouped into upper-level nonmanual workers (n = 1886, 10.7%, comprising managers and/or professionals) and others (n = 15,754, 89.3%), according to previous studies^6,11^. In general, regarding socioeconomic disparities in mortality, upper-level nonmanual workers, including individuals with the highest occupational class, tend to have the best advantages in economic (income, security, and advancement prospects) and occupational dimensions (authority and autonomy)^6,11^.

### Remote work and other covariates

Remote work was considered a primary mediating variable that may influence changes in occupational CHD risk. Participants were categorized as engaged in remote work if they reported working from home at least once per month during the baseline assessment.

When examining the association between occupational class and CHD risk, the following fundamental confounding variables were considered: sex, age, educational attainment (≤ 12 years [high school] or ≥ 13 years [college or university]), and household income (< 2 million JPY [approximately 20,000 USD] or ≥ 2 million JPY). Additionally, to further consider potential mediators, various covariates of behavioral factors (smoking, habitual drinking [> 1 day/week], and physical activity [either a ≥ 30 min light sweating exercise more than once per week or a ≥ 1 h walking or equivalent activities more than once per week]), clinical factors (body mass index, hypertension, and diabetes), psychological distress (Kessler 6 scores > 10), and long working hours (> 55 h weekly) were included^23^.

### Statistical analysis

Risk ratios (RRs) and 95% confidence intervals (CIs) were estimated for CHD incidence among upper-level nonmanual workers. The other-occupation group served as the reference group. A two-level multilevel Poisson regression with robust variance, where participants (Level 1) were nested within 47 prefectures (Level 2), was employed, and a random intercept for prefectures was applied^24^. Owing to the lack of precise timing information for CHD onset in the study, incidence rate ratios could not be estimated. In the multivariable regression analysis, adjustments were made for basic confounding variables of sex, age, educational attainment, and household income (Model 1). In Model 2, a primary mediating variable of remote work was added. Finally, all potential mediating variables were accounted for in Model 3. Moreover, in Models 2 and 3, the estimated RRs of remote work were acknowledged as explanation factors for CHD incidence related to remote work. In a subgroup analysis, stratified analyses were conducted by age groups: (i) a younger population aged 20–49 years and (ii) an older population aged 50–65 years. Sex-specific analyses were also performed.

**Figure 2:**
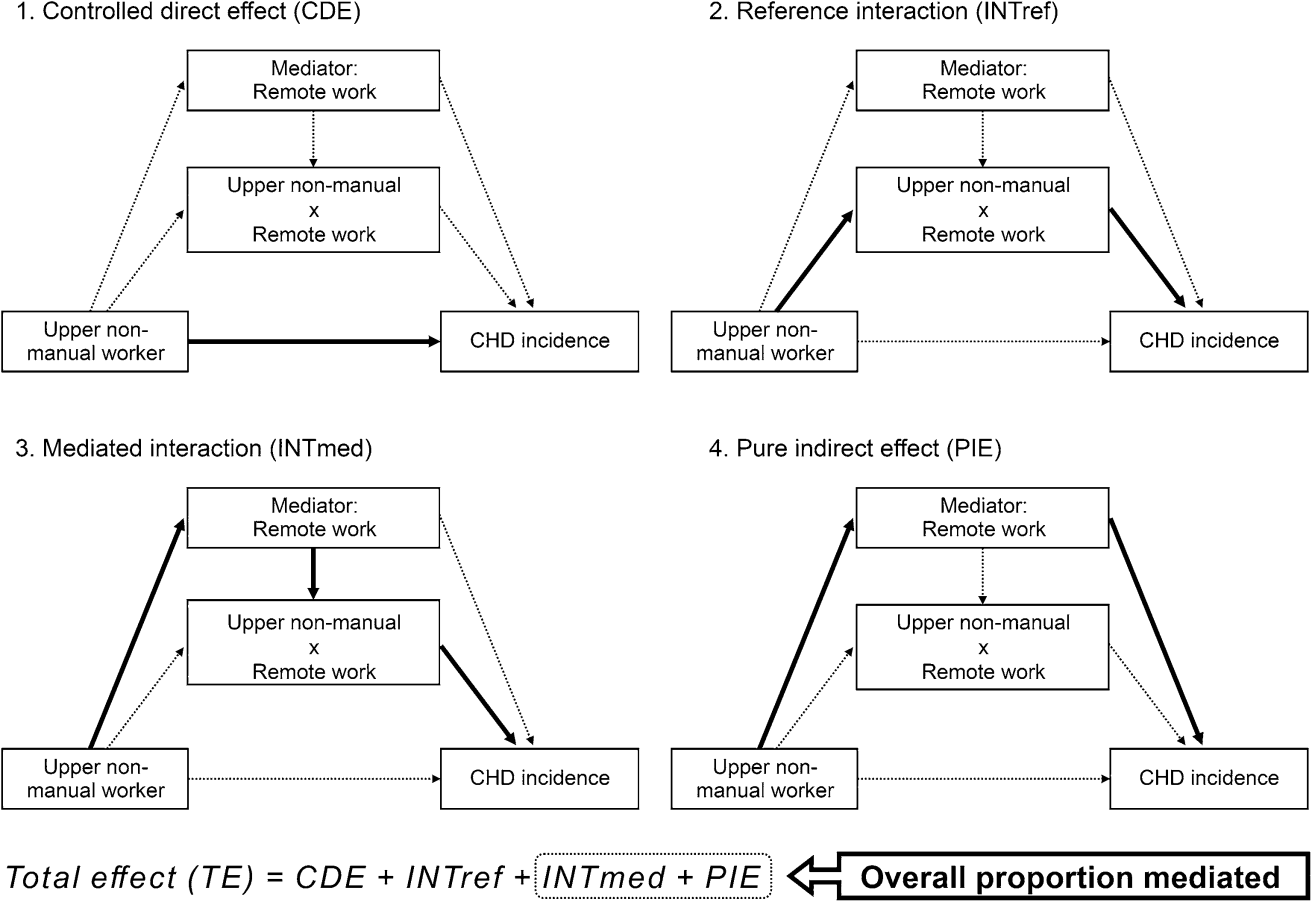
Causal diagram representing the four-way decomposition of the association between upper-level nonmanual workers, remote work, and coronary heart disease incidence. The exposure (upper-level nonmanual worker), mediator (remote work), confounders (sex, age, educational attainment, and household income), and outcome (coronary heart disease [CHD] incidence) were defined. Logistic regression models were fitted with a four-way decomposition for the outcome (as a function of the exposure, the mediator, exposure-mediator interaction, and confounders) and the mediator (as a function of the exposure and confounders). The total effect (TE) of the exposure, in the presence of the mediator with which the exposure may interact, on the outcome is decomposed into four components due to just mediation (pure indirect effect, PIE), just interaction (reference interaction, INTref), both mediation and interaction (mediated interaction, INTmed), and neither mediation nor interaction (controlled direct effect, CDE): i.e., TE = CDE + INTref + INTmed + PIE. The overall proportion of the total effect explained by the mediating effect (“total indirect effect”) was estimated using these four components, as follows: (INTmed + PIE)/TE.

Furthermore, a causal mediation analysis (Fig. 2) using a four-way decomposition method was performed to estimate the proportion of occupational disparities in CHD risk in upper-level nonmanual workers explained by remote work^25^. The exposure (upper-level nonmanual worker), mediator (remote work), confounders (sex, age, educational attainment, and household income), and outcome (CHD incidence) were defined^24–27^. Statistical significance was set at 0.05, and all *P*-values were two-sided. Data were analyzed using STATA/MP17 (StataCorp LLC, College Station, TX, USA).

### Supplementary Information

Supplementary Information.

## Data Availability

The datasets generated during and/or analyzed during the current study are available from the corresponding author on reasonable request.
