# Supplementary material for: Incidence of coronary heart disease among remote workers: a nationwide web-based cohort study
Source: Sci Rep. 2024 Apr 10;14:8415. doi: 10.1038/s41598-024-59000-y (PMC11006843; doi:10.1038/s41598-024-59000-y)
Supplement: Supplementary file 1 — Supplementary Information. [file 41598_2024_59000_MOESM1_ESM.pdf]

Incidence of coronary heart disease among remote workers: A nationwide web-based cohort study

Masayoshi Zaitsu, Tomohiro Ishimaru, Saki Tsushima, Keiji Muramatsu, Hajime Ando, Tomohisa Nagata, Hisashi Eguchi, Seiichiro Tateishi, Mayumi Tsuji, Yoshihisa Fujino; for the CORoNa Work Project

Supplemental Material

Table S1–S3

Table S1. Relative risks for coronary heart disease incidence estimated by two-level multilevel Poisson regression with robust variance

**Characteristics**	Relative risk (95% confidence interval)[a]		
	Model 1	Model 2	Model 3
Entire population (age 20–65 years)			
Upper-level nonmanual worker	1.12 (0.82–1.53)	1.07 (0.78–1.47)	1.07 (0.77–1.47)
Female	0.45 (0.35–0.59)	0.46 (0.35–0.59)	0.54 (0.40–0.73)
Age	1.01 (0.99–1.02)	1.00 (0.98–1.02)	1.00 (0.98–1.02)
High school or less	1.17 (0.88–1.55)	1.24 (0.93–1.66)	1.19 (0.88–1.62)
Low household income <2 million JPY	1.49 (0.87–2.54)	1.42 (0.83–2.43)	1.36 (0.80–2.33)
Remote work at baseline		1.50 (1.20–1.89)	1.49 (1.19–1.86)
Ever smoker			1.26 (0.93–1.69)
Habitual drinker >1 day/week			1.23 (0.96–1.56)
Physically active			1.34 (1.03–1.75)
Body mass index			1.01 (0.98–1.04)
Hypertension			1.64 (1.19–2.28)
Diabetes			1.31 (0.84–2.05)
Kessler 6 scores >10			2.21 (1.75–2.78)
Weekly working hours >55 h			0.91 (0.67–1.23)
Younger population (20–49 years)			
Upper-level nonmanual worker	2.19 (1.40–3.42)	1.96 (1.24–3.08)	1.88 (1.18–3.00)
Female	0.40 (0.28–0.57)	0.41 (0.29–0.59)	0.51 (0.34–0.76)
Age	0.97 (0.94–1.00)	0.97 (0.94–1.00)	0.96 (0.93–0.99)
High school or less	1.26 (0.76–2.09)	1.38 (0.85–2.26)	1.30 (0.79–2.14)
Low household income <2 million JPY	1.44 (0.57–3.61)	1.31 (0.50–3.41)	1.24 (0.48–3.21)
Remote work at baseline		2.10 (1.51–2.91)	1.92 (1.37–2.68)

Ever smoker			1.42 (0.86–2.35)
Habitual drinker >1 day/week			1.52 (0.99–2.35)
Physically active			1.84 (1.32–2.58)
Body mass index			1.02 (0.97–1.06)
Hypertension			2.85 (1.79–4.56)
Diabetes			0.51 (0.13–1.99)
Kessler 6 scores >10			2.39 (1.71–3.34)
Weekly working hours >55 h			0.53 (0.25–1.13)
Older population (50–65 years)			
Upper-level nonmanual worker	0.87 (0.63–1.21)	0.86 (0.61–1.21)	0.87 (0.62–1.23)
Female	0.49 (0.32–0.76)	0.50 (0.32–0.76)	0.58 (0.36–0.94)
Age	1.05 (1.01–1.09)	1.05 (1.01–1.09)	1.05 (1.01–1.09)
High school or less	1.17 (0.75–1.81)	1.20 (0.77–1.86)	1.17 (0.75–1.83)
Low household income <2 million JPY	1.46 (0.83–2.58)	1.43 (0.82–2.50)	1.36 (0.78–2.37)
Remote work at baseline		1.19 (0.86–1.66)	1.21 (0.88–1.67)
Ever smoker			1.13 (0.79–1.62)
Habitual drinker >1 day/week			1.02 (0.78–1.34)
Physically active			1.04 (0.75–1.44)
Body mass index			1.02 (0.97–1.07)
Hypertension			1.31 (0.91–1.90)
Diabetes			1.46 (0.88–2.42)
Kessler 6 scores >10			2.11 (1.56–2.85)
Weekly working hours >55 h			1.18 (0.81–1.72)

[a] Participants (Level 1) were nested within 47 prefectures (Level 2), and a random intercept was employed for prefectures.

Table S2. Results of causal mediation analysis and four-way decomposition for all and older populations

Four-way decomposition	Coefficient (95% CI)	
	Entire population 20–65 years	Older population 50–65 years
Total excess odds ratio	0.12 (-0.26–0.49)	-0.13 (-0.50–0.23)
Excess odds ratio due to CDE	0.03 (-0.39–0.44)	-0.11 (-0.55–0.34)
Excess odds ratio due to INTref	0.03 (-0.14–0.20)	-0.03 (-0.22–0.16)
Excess odds ratio due to INTmed	0.02 (-0.07–0.10)	-0.01 (-0.09–0.07)
Excess odds ratio due to PIE	0.05 (0.01–0.08)	0.02 (-0.02–0.06)
Total effect	1.12 (0.74–1.49)	0.87 (0.50–1.23)
Proportion CDE	0.21 (-2.80–3.23)	0.81 (-1.16–2.79)
Proportion INTref	0.26 (-1.31–1.83)	0.23 (-1.33–1.80)
Proportion INTmed	0.13 (-0.67–0.94)	0.10 (-0.58–0.78)
Proportion PIE	0.40 (-0.90–1.70)	-0.15 (-0.67–0.37)
Overall proportion mediated	0.53 (-1.18–2.24)	-0.05 (-0.60–0.51)
Overall proportion attributable to interaction	0.39 (-1.98–2.76)	0.34 (-1.91–2.58)
Overall proportion eliminated	0.79 (-2.23–3.80)	0.19 (-1.79–2.16)

Table S3. Profiles of 920 excluded participants with a medical history of CHD at baseline

Characteristics	N (%) or mean (SD)
N	920
Upper-level non-manual	93 (10.1%)
Remote work at baseline	203 (22.1%)
Female	366 (39.8%)
Age, mean (SD)	49.0 (9.9)
High school or less	263 (28.6%)
Low household income <2 million JPY	101 (11.0%)
Ever smoker	502 (54.6%)
Former smoker	217 (23.6%)
Current smoker	285 (31.0%)
Habitual drinker >1 day/week	365 (39.7%)
Physically active	350 (38.0%)
Body mass index, mean (SD)	22.9 (4.2)
Hypertension	827 (89.9%)
Diabetes	784 (85.2%)
Kessler 6 scores >10	426 (46.3%)
Weekly working hours >55 h	133 (14.5%)
